# Predictive control strategy of a gas turbine for improvement of combined cycle power plant dynamic performance and efficiency

**DOI:** 10.1186/s40064-016-2679-2

**Published:** 2016-07-04

**Authors:** Omar Mohamed, Jihong Wang, Ashraf Khalil, Marwan Limhabrash

**Affiliations:** 1 Department of Electrical Engineering, Princess Sumaya University for Technology, P.O. Box 1438, Al-Jubaiha, 11941 Jordan; 2 School of Engineering, University of Warwick, Coventry, CV4 7AL UK; 3 Department of Electrical Engineering, University of Benghazi, P.O. Box 9476, Benghazi, Libya; 4 Production Department, General Electric Company of Libya, Tripoli, Libya

**Keywords:** Gas turbines, Model predictive control (MPC), Natural gas industry, Subspace identification

## Abstract

This paper presents a novel strategy for implementing model predictive control (MPC) to a large gas turbine power plant as a part of our research progress in order to improve plant thermal efficiency and load–frequency control performance. A generalized state space model for a large gas turbine covering the whole steady operational range is designed according to subspace identification method with closed loop data as input to the identification algorithm. Then the model is used in developing a MPC and integrated into the plant existing control strategy. The strategy principle is based on feeding the reference signals of the pilot valve, natural gas valve, and the compressor pressure ratio controller with the optimized decisions given by the MPC instead of direct application of the control signals. If the set points for the compressor controller and turbine valves are sent in a timely manner, there will be more kinetic energy in the plant to release faster responses on the output and the overall system efficiency is improved. Simulation results have illustrated the feasibility of the proposed application that has achieved significant improvement in the frequency variations and load following capability which are also translated to be improvements in the overall combined cycle thermal efficiency of around 1.1 % compared to the existing one.

## Background

In recent years, gas turbines (GT) have easily reached a primary position in thermal power generation field because of their fast deliveries of power and availability of natural gas (NG) (Rayaprolu 2009). GT as simple cycle or integrated with heat recovery steam generator (HRSG) to form a combined cycle (CC) power plant has become very popular generation technology in many countries. However, the manufacturers of such devices are doing great effort to achieve improved efficiencies and lower pollutant emissions that compete coal and clean coal technologies. Nowadays, apart from reliability and fuel cost optimization, novel power generation techniques demand much improved load demand tracking that lead to lessen frequency variations of power system with better corresponding plant efficiency. It has been noticed that improving the operation performance of gas turbine can significantly lead to higher cycle efficiency and better dynamic performance. Achieving better compressing ratios and maintaining the exhausted gas temperature within certain limits despite load stochastic variations is likely to do just that improvement. In the developed countries, it is, however, reported that the efficiency of CC power plants from 1960 to 2000 has been improved from 35 % up to nearly 60 % respectively (Rayaprolu 2009). In general, the literature of thermal power plants has often suggested optimal and predictive control theories that meet wide acceptance in industry and power plants (Lee and Ramirez 2001). In particular, some articles has been written and published on CC power plants’ control that sought to optimize combined cycle power plants with regard to efficiency and load following capability (Saez et al. 2007; Matsumoto et al. 1996; Lalor et al. 2005). A fuzzy predictive control based on genetic algorithms for power plant gas turbines is developed which provides the optimal dynamic set point for the regulatory level with contribution to capturing the nonlinearity of the plant (Saez et al. 2007). Start-up process optimization by an expert system has been proposed under NO_x_ emission regulation and management of machine life (Matsumoto et al. 1996). The influence of gas turbine short-term dynamics on the performance of frequency control can also be investigated through suitable modelling for knowledge of frequency excursions in the grid in advance (Lalor et al. 2005). However, there are other important factors that have a direct influence on the system output performance and the grid frequency, which are studied in this paper and not considered in many papers. For instance, in the previous literature the emphasis of fuel flow changes was given only to the valve position of natural gas (NG); however, the position of pilot gas valve can also be manipulated to stabilize the flames in the premix and ensure steady combustion at all time. In addition, the compressor pressure ratio signal (the ratio of the discharged pressure of the compressor to the inlet atmospheric pressure) passes through the compressor pressure limit controller to influence the inlet guide vane (IGV) pitch controller which in turn affects the combustion process and flow of air to the GT. If the system automation is upgraded with potential to correct such signals by the MPC, these signals will optimize in advance which means reduced process variations while keeping faster load following capability due to higher stored/kinetic energy in the plant. This also can make the CCGT works close to its optimum efficiency by expanding the pressure ratio and the firing temperature by MPC. These potent motivations have to be investigated on gas turbines through advanced technique of system identification and control system design. However, without digital simulation, the gap between theory and practice cannot be easily bridged in this task. A model has been developed and verified via identification technique which is assessed and published in our research (Mohamed et al. 2015). The scientific contribution added to this paper is to integrate MPC to the developed identified model representing the real GT with emphasis on the strategic influences discussed above for the target of performance enhancement. The present paper is organized as follows. “Combined (deterministic/stochastic) subspace identification” section clarifies the technique of subspace identification. “The application of subspace identification to gas turbine process” section discusses the developed model of real gas turbine by subspace identification method for different operating regions. Simulation results of identification and verification procedure have shown model accuracy and capability of reflecting the key variables of the turbine. “Predictive controller design and implementation” section presents the designed model predictive control to be applied to the system. “Simulation results” section then mentions simulation scenarios that have offered the advantage of the proposed upgraded strategy. Finally, the present paper has concluded the work with suggested further opportunities for future research.

## Combined (deterministic/stochastic) subspace identification

### Theoretical foundation for subspace identification

Some applied linear algebra may be necessary to simplify the description of subspace identification method. Subspace identification is based on the tools of singular value decomposition and oblique projection. The reader is highly recommended to refer to the text (Meyer 2000) for more details.

#### Singular value decomposition

Singular value decomposition (SVD) is a matrix analysis that facilitates the subspace identification method. It simply states that an *m* × *n* matrix **M** could be dissected into three matrices, two of them are orthogonal matrices and one is a diagonal matrix contains the singular values of the main matrix as nonzero diagonal elements. Though it is applied on either real or complex matrix, it is assumed in our application that the matrices are real. Then we have for every **M** $\in$ *R* ^*m*×*n*^ of rank r, there are orthogonal matrices **U**_*m*×*m*_, **V**_*n*×*n*_ and a diagonal matrix **S**_*r*×*r*_ = dig (σ_1_, σ_2_, o_*r*_) such that

1 $${\mathbf{M}} = {\mathbf{U}}\left( {\begin{array}{*{20}l} {\mathbf{S}} \hfill &\quad {\mathbf{0}} \hfill \\ {\mathbf{0}} \hfill &\quad {\mathbf{0}} \hfill \\ \end{array} } \right)_{{{\mathbf{n}} \times {\mathbf{m}}}} {\mathbf{V}}^{{\mathbf{T}}}$$

The factorization in Eq. (1) is known as singular value decomposition of **M**. The columns in **U** and **V** are called the left hand and right hand singular vectors of **M**, respectively. For matrix computations and analysis refer to Meyer (2000) and Mohamed et al. (2014).

#### Orthogonal projection and oblique projection

Suppose that we have the subspaces $\mathcal{V}$ and $\mathcal{W},$ then, the orthogonal projection of the row space of $\mathcal{V}$ into the row space of $\mathcal{W}$ is formulated as follows (Meyer 2000; Ruscio 2009; Overschee and Moore 1996):

2 $$\mathcal{V} /\mathcal{W} = \mathcal{ V W}^{{{\dag }}} \mathcal{W }$$

where † stands for the Moore–Penrose pseudo-inverse that facilitates the concept of orthogonal projection of the matrix which is defined as

3 $$\mathcal{W}^{{\dag }} = (\mathcal{W}^{\rm T} \mathcal{W})^{{{ - 1}}} \mathcal{W}^{\rm T}$$

Oblique projection of row space of matrix $\mathcal{V}$ onto the row space of matrix $\mathcal{M}$ along the row space of matrix $\mathcal{W}$ can be defined as

4 $$\mathcal{V} /_{\mathcal{W}} \mathcal{M} = \left[ {\mathcal{V} /\mathcal{W}^{ \bot } } \right] \cdot \left[ {\mathcal{M} /\mathcal{W}^{ \bot } } \right]^{{{\dag }}} \cdot \mathcal{M}$$

where $\mathcal{W}^{ \bot }$ is the orthogonal projection into the null space of $\mathcal{W}$ such that $\mathcal{W}^{ \bot } \cdot \mathcal{W} = 0.$ In identification of combined systems, the identification of the deterministic part is done by means of projection and singular value decomposition (Meyer 2000). In general, an instrument matrix is multiplied by both sides of the extended state space model to remove the stochastic part and the input vector so that we can get the extended observability matrix and state sequence. Once the extended observability matrix is known, the system matrices can be found. This is discussed in details in the next section.

### The subspace identification technique

This section presents the algorithm of subspace identification method. The method has emerged in late 1980s and resolved many problems regarding identification of complex industrial processes (Ruscio 2009; Overschee and Moore 1996). It has been proved that it is capable of identifying the key features of gas turbine power plants (Mohamed et al. 2014). The method of subspace identification is based on the advanced matrix linear algebra techniques which are singular value decomposition and oblique projection. The problem is described as follows (Ruscio 2009; Overschee and Moore 1996).

A set of data measured for combined unknown system of order n:

5 $$x_{k + 1} = Ax_{k} + Bu_{k} + w_{k}$$

6 $$y_{k} = Cx_{k} + Du_{k} + v_{k}$$

With *w* and *v* are zero mean white noise innovations with covariance matrix

$${\mathbf{E}}\left[ {\left( {\begin{array}{*{20}l} {w_{p} } \\ {v_{p} } \\ \end{array} } \right)\left( {\begin{array}{*{20}l} {w_{p}^{T} } &\quad {v_{p}^{T} } \\ \end{array} } \right)} \right] = \left( {\begin{array}{*{20}l} Q \hfill &\quad S \hfill \\ {S^{T} } \hfill &\quad R \hfill \\ \end{array} } \right)\delta_{pq}$$

With knowledge of system inputs/outputs *u*_*k*_ and *y*_*k*_, the problem is to determine/identify:

1. The system order n.

2. The system matrices $A \in R^{n \times n} ,\,B \in R^{n \times m} ,C \in R^{l \times n} ,\,D \in R^{l \times m}$ and the matrices, $Q \in R^{n \times n} ,\,S \in R^{n \times l} ,\, \in R^{l \times l}$ so that the model output agree with the main variation trends of the output data. The system extended state space model can be organized as follows:

7 $$Y_{f} = O_{i} X_{f} + H_{i}^{d} U_{f} + H_{i}^{s} E_{f} + N_{f}$$

where *Y*_*f*_, *U*_*f*_, *X*_*f*_, *E*_*f*_, *N*_*f*_ denotes the future output, future input, future states, and future noises. The matrices are defined as follows:

$$\begin{aligned} & O_{i}\, \mathop = \limits^{def}\, \left[ {\begin{array}{*{20}l} C \\ {CA} \\ \vdots \\ {CA^{i - 1} } \\ \end{array} } \right] \in R^{im \times n} ,\quad H_{i}^{d}\, \mathop = \limits^{def}\, \left[ {\begin{array}{*{20}l} D &\quad 0 &\quad 0 &\quad \cdots &\quad 0 \\ {CB} &\quad D &\quad 0 &\quad \cdots &\quad 0 \\ {CAB} &\quad {CB} &\quad D &\quad \cdots &\quad 0 \\ \vdots &\quad \vdots &\quad \vdots &\quad \ddots &\quad \vdots \\ {CA^{i - 2} B} &\quad {CA^{i - 3} B} &\quad {CA^{i - 4} B} &\quad \cdots &\quad D \\ \end{array} } \right], \\ & H_{i}^{s}\, \mathop = \limits^{def}\,\left[ {\begin{array}{*{20}l} 0 &\quad 0 & \quad 0 &\quad \cdots &\quad 0 \\ C &\quad 0 &\quad 0 &\quad \cdots &\quad 0 \\ {CA} &\quad C &\quad 0 &\quad \cdots &\quad 0 \\ \vdots &\quad \vdots &\quad \vdots &\quad \ddots &\quad \vdots \\ {CA^{i - 2} } &\quad {CA^{i - 3} } &\quad {CA^{i - 4} } &\quad \cdots &\quad 0 \\ \end{array} } \right] \\ \end{aligned}$$

*H*_*i*_^*d*^ is known as deterministic Toeplitz matrix while *H*_*i*_^*s*^ is the stochastic Toeplitz matrix. The data is sampled and organized as Hankel matrix, the input data matrix for past and future samples

$$U_{\left. 0 \right|2i - 1}\, \mathop = \limits^{def}\, \left( {\frac{{\begin{array}{*{20}l} {u_{0} } &\quad {u_{1} } &\quad {u_{2} } &\quad \cdots &\quad {u_{j - 1} } \\ {u_{1} } &\quad {u_{2} } &\quad {u_{3} } &\quad \cdots &\quad {u_{j} } \\ \cdots &\quad \cdots &\quad \cdots &\quad \cdots &\quad \cdots \\ {u_{i - 1} } &\quad {u_{i} } &\quad {u_{i + 1} } &\quad \cdots &\quad {u_{i + j - 2} } \\ \end{array} }}{{\begin{array}{*{20}l} {u_{i} } &\quad {u_{i + 1} } &\quad {u_{i + 2} } &\quad \cdots &\quad {u_{i + j - 1} } \\ {u_{i + 1} } &\quad {u_{i + 2} } &\quad {u_{i + 3} } &\quad \cdots &\quad {u_{i + j} } \\ \cdots &\quad \cdots &\quad \cdots &\quad \cdots &\quad \cdots \\ {u_{2i - 1} } &\quad {u_{2i} } &\quad {u_{2i + 1} } &\quad \cdots &\quad {u_{2i + j - 2} } \\ \end{array} }}} \right)\mathop = \limits^{def} \left( {\frac{{U_{p} }}{{U_{f} }}} \right)$$

and the output data matrix is

$$Y_{\left. 0 \right|2i - 1} \mathop = \limits^{def} \left({\frac{{\begin{array}{*{20}l} {y_{0} } &\quad {y_{1} } &\quad {y_{2} }&\quad \cdots &\quad {y_{j - 1} } \\ {y_{1} } &\quad {y_{2} } &\quad{y_{3} } &\quad \cdots &\quad {y_{j} } \\ \cdots &\quad \cdots &\quad\cdots &\quad \cdots &\quad \cdots \\ {y_{i - 1} } &\quad {y_{i} }&\quad {y_{i + 1} } &\quad \cdots &\quad {y_{i + j - 2} } \\ \end{array} }}{{\begin{array}{*{20}l} {y_{i} } &\quad {y_{i + 1} } &\quad {y_{i + 2} } &\quad \cdots &\quad {y_{i + j - 1} } \\ {y_{i + 1} } &\quad {y_{i + 2} } &\quad {y_{i + 3} } &\quad \cdots &\quad {y_{i + j} } \\ \cdots &\quad \cdots &\quad \cdots &\quad \cdots &\quad \cdots \\ {y_{2i - 1} } &\quad {y_{2i} } &\quad {y_{2i + 1} } &\quad \cdots &\quad {y_{2i + j - 2} } \\ \end{array} }}} \right)\mathop = \limits^{def} \left( {\frac{{Y_{p} }}{{Y_{f} }}} \right)$$

where the subscript *p* and *f* denote the past and future respectively. The same can be done for matrix *E*_*i*_. The state vector *X*_*i*_ is defined as

$$X_{i}\, \mathop = \limits^{def}\, \left( {\begin{array}{*{20}l} {x_{i} } &\quad {x_{i + 1} } &\quad {x_{i + 2} } &\quad \ldots &\quad {x_{i + j - 1} } \\ \end{array} } \right).$$

### Proof of extended state space model

Looking at the general state space model in (5) and (6). The extended state space model that contains the matrices data can be easily derived;

8 $$y_{k + 1} = Cx_{k + 1} + Du_{k + 1} + v_{k + 1}$$

Substitute (5) in (8) we get

$$y_{k + 1} = CAx_{k} + CBu_{k} + Cw_{k} + Du_{k + 1} + v_{k + 1}$$

Since

9 $$y_{k + 2} = Cx_{k + 2} + Du_{k + 2} + v_{k + 2}$$

and

10 $$x_{k + 2} = Ax_{k + 1} + Bu_{k + 1} + w_{k + 1}$$

Then from (9) and (10) we get,

11 $$y_{k + 2} = CAx_{k + 1} + CBu_{k + 1} + Cw_{k + 1} + Du_{k + 2} + v_{k + 2}$$

Substituting (5) in (11), we get:

$$\begin{aligned} y_{k + 2} & = CA^{2} x_{k} + CABu_{k} + CAw_{k} + CBu_{k + 1} \\ & \quad + Cw_{k + 1} + Du_{k + 2} + v_{k + 2} \\ \end{aligned}$$

Organizing the above equations as matrix equation; with extended data vectors *y, u*, and *v*

12 $$\left[ {\begin{array}{*{20}l} {y_{k} } \\ {y_{k + 1} } \\ {y_{k + 2} } \\ \end{array} } \right] = \left[ {\begin{array}{*{20}l} C \\ {CA} \\ {CA^{2} } \\ \end{array} } \right]x_{k}\,+ \left[ {\begin{array}{*{20}l} D \hfill &\quad 0 \hfill &\quad 0 \hfill \\ {CB} \hfill &\quad D \hfill &\quad 0 \hfill \\ {CAB} \hfill &\quad {DB} \hfill &\quad D \hfill \\ \end{array} } \right]\left[ {\begin{array}{*{20}l} {u_{k} } \\ {u_{k + 1} } \\ {u_{k + 2} } \\ \end{array} } \right] + \left[ {\begin{array}{*{20}l} 0 \hfill &\quad 0 \hfill &\quad 0 \hfill \\ C \hfill &\quad 0 \hfill &\quad 0 \hfill \\ {CA} \hfill &\quad C \hfill &\quad 0 \hfill \\ \end{array} } \right]\left[ {\begin{array}{*{20}l} {w_{k} } \\ {w_{k + 1} } \\ {w_{k + 2} } \\ \end{array} } \right] + \left[ {\begin{array}{*{20}l} {v_{k} } \\ {v_{k + 1} } \\ {v_{k + 2} } \\ \end{array} } \right]$$

For *i* instants (block rows) and *j* number of experiments (block columns), we get Eq. (12) with the, inputs, outputs and states defined;

13 $$Y_{f} = O_{i} X_{f} + H_{i}^{d} U_{f} + H_{i}^{s} E_{f} + N_{f}$$

14 $$Y_{p} = O_{i} X_{p} + H_{i}^{d} U_{p} + H_{i}^{s} E_{p} + N_{p} .$$

Subspace identification algorithms **N4SID** which stands for **N**umerical algorithm for ***S***ubspace ***S***tate ***S***pace ***S***ystem ***Id***entification (Ruscio 2009). We shall now define the block Hankel matrix that contains the past inputs and outputs *W*_*p*_

$$W_{p} = \left( {\frac{{U_{p} }}{{Y_{p} }}} \right),$$

The general steps for subspace identification are (Ruscio 2009; Overschee and Moore 1996):

1. Calculate the oblique projection:

This algorithm is based on oblique projection and singular value decomposition. The tool of oblique projection is mainly used to extract the term of extended observability matrix and the sequence of states [i.e. the term *O*_*i*_ *X*_*f*_ in (7)]. Projection of row space of future output *Y*_*f*_ onto *W*_*p*_ along the future input *U*_*f*_

$$\zeta_{i} = Y_{f} /_{{U_{f} }} W_{p} \quad {\text{and}}\quad \zeta_{i + 1} = Y_{f}^{ + } /_{{U_{f}^{ + } }} W_{p}^{ + }$$

From (8)

$$Y_f/U_f\,\,W_{p} = \left[ {Y_{f} /U_{f}^{ \bot } } \right] \cdot \left[ {W_{p} /U_{f}^{ \bot } } \right]^{{^{{\dag }} }} W_{p}$$

where $U_{f}^{ \bot }$ is the orthogonal complement of the raw space of $U_{f} .$ According to the elementary linear algebra given in (Overschee and Moore 1996),

$$\begin{aligned} Y_{f} /U_{f} & = Y_{f} U_{f}^{{\dag }} U_{f} \\ Y_{f} /U_{f}^{ \bot } & = Y_{f} - Y_{f} /U_{f} \\ \end{aligned}$$

There are weighting matrices *W*_1_ and *W*_2_ to be multiplied by the oblique projection to remove the stochastic part (i.e. $W_{1} \cdot (H_{i}^{s} M_{i} + N_{i} ) \cdot W_{2} = 0$). The choice of these matrices is relatively arbitrary and different from one algorithm to another (Ruscio 2009; Overschee and Moore 1996). However, they are chosen to satisfy the equation mentioned.

2. Calculate the singular value decomposition SVD of weighted oblique projection:
  15 $$W_{1} \xi_{i} W_{2} = USV^{T} = \left( {\begin{array}{*{20}l} {U_{1} } &\quad {U_{2} } \\ \end{array} } \right)\left( {\begin{array}{*{20}l} {S_{1} } &\quad 0 \\ 0 &\quad 0 \\ \end{array} } \right)\left( {\begin{array}{*{20}l} {V_{1}^{T} } \\ {V_{2}^{T} } \\ \end{array} } \right)$$
3. Estimate the system order by counting the nonzero singular values of S and set apart the SVD to obtain *U*_1_ and *S*_1_.
4. Calculate the extended observability matrix *O*_*i*_ and *O*_*i*−1_ from:
  16 $$O_{i} = W_{1}^{ - 1} U_{1} S_{1}^{1/2}$$
5. Determine the sequences of states *X*_*i*_ and *X*_*i*+1_
  $$\begin{aligned} \tilde{X}_{i} & = O_{i}^{{{\dag }}} \zeta_{i} \\ \tilde{X}_{i + 1} & = O_{i - 1}^{{{\dag }}} \zeta_{i + 1} \\ \end{aligned}$$

The superscript † means the Moore–Penrose pseudoinverse.

6. Up to this step, the system states are known with the system inputs/outputs processed data. Then, solve the following linear equation for the system matrices *A*, *B*, *C* and *D.*
  17 $$\left( {\begin{array}{*{20}l} {\tilde{X}_{i + 1} } \\ {Y_{i\left| i \right.} } \\ \end{array} } \right) = \left( {\begin{array}{*{20}l} A &\quad B \\ C &\quad D \\ \end{array} } \right)\left( {\begin{array}{*{20}l} {\tilde{X}_{i} } \\ {U_{i\left| i \right.} } \\ \end{array} } \right) + \left( {\begin{array}{*{20}l} {\rho_{w} } \\ {\rho_{v} } \\ \end{array} } \right)$$
7. For stochastic part, estimate Q, R, and S from the residuals:
  18 $$\left( {\begin{array}{*{20}l} Q \hfill &\quad S \hfill \\ {S^{T} } \hfill &\quad D \hfill \\ \end{array} } \right) = {\mathbf{E}}_{{\mathbf{j}}} \left[ {\left( {\begin{array}{*{20}l} {\rho_{w} } \\ {\rho_{v} } \\ \end{array} } \right) \cdot \left( {\begin{array}{*{20}l} {\rho_{w}^{T} } & {\rho_{v}^{T} } \\ \end{array} } \right)} \right]$$
  For more details about subspace identification method, refer to Overschee and Moore (1996).

## The application of subspace identification to gas turbine process

**Fig. 1:**
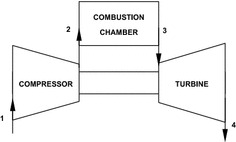
Gas turbine

This section discusses the process of gas turbine technology, the preparation of data signals, and the simulation results for the method of subspace technique for both phases of research (IEEE Power System Dynamic Performance Committee 2013; Modau and Pourbeik 2008). However, the need for developing gas turbine model by alternative advanced techniques is one of the main strong motivations behind this paper. The main components of gas turbine are shown in Fig. 1, a compressor, a combustion chamber, and a turbine. The air required for combustion is supplied by the compressor (process 1–2); there in the combustion chamber the air is mixed with the fuel and combusted (process 2–3). In ideal situations, the process 1–2 is an isentropic process while process 2–3 is isobaric or constant pressure process. The expansion of the hot combusted gases in the turbine is an isentropic (process 3–4) which produces useful work in the turbine sufficient to derive the rotor of the synchronous generator. Finally, heat rejection process takes place at constant pressure (process 4–1). The exhausted gas from the gas turbine is used to energize the HRSG to supply a steam turbine with the necessary superheated steam. The remaining electricity is produced by the generator which is mechanically coupled to steam turbine supplied by the HRSG (IEEE Power System Dynamic Performance Committee 2013; Sonntag and Borgnakke 1998).

**Fig. 2:**
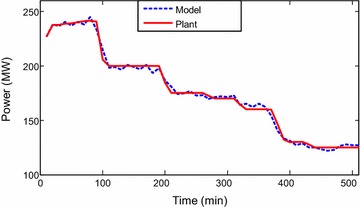
Identification of power output signal

**Fig. 3:**
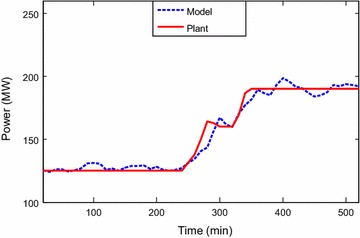
Verification of output power signal

**Fig. 4:**
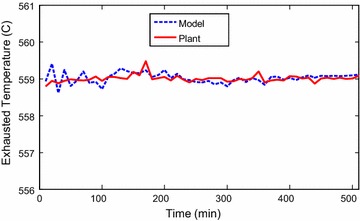
Identification of exhausted temperature signal

**Fig. 5:**
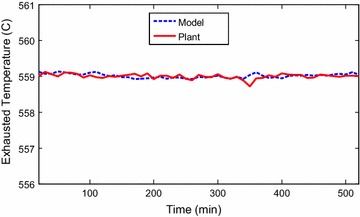
Verification of exhausted temperature signal

**Fig. 6:**
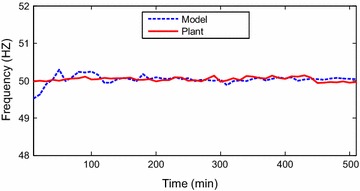
Identification of grid frequency signal

**Fig. 7:**
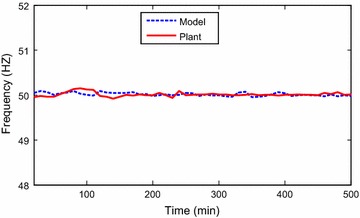
Verification of grid frequency signal

The data points were collected as discrete time signals from the industrial team at the General Electric Company of Libya at the plant centre of control at North Benghazi Power Plant in the eastern part of Libya. Three sets of data were organized. One set of data is used for identification phase and the other two sets of data are used for verification. The inputs to the system to be identified, from control point of view, have been selected to be the natural gas (NG) control valve (%), the pilot gas valve (%), and the compressor outlet pressure (bar). These are regarded later to be the manipulated inputs of the MPC to be fed as set points to the subsystems of the process. The output signals are the power output (MW) of the turbine, the exhausted temperature of the turbine (C^Ɵ^), and the frequency of the grid (Hz). System Identification toolbox has been utilized (Ljung 2010). Identification and sample of verification results are presented through Figs. 2, 3, 4, 5, 6 and 7. The model responses nicely agree with the main trends of the real plant responses. The model parameters appear in the “Appendix”.

## Predictive controller design and implementation

### Description of a portion in the current automation system

**Fig. 8:**
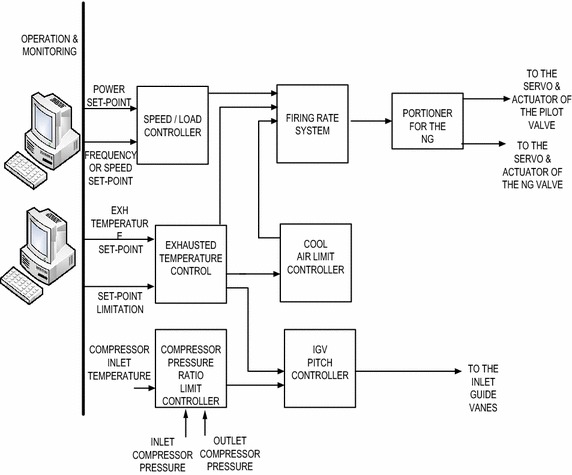
A portion of interest of the GT control system at North Benghazi Power Plant

The concept proposed in this work is applied to a specific portion of the existing control unit, which is responsible for the variables of interests. The current situation of the GT from control point of view is investigated through site visits and plant operation documentation (Daewoo E&C, Siemens 2009 Approval). A functional blocks diagram that shows the critical components for the control system to be upgraded is shown in Fig. 8. It should be mentioned that there are lots of other control circuits that performs other tasks of control, but this research considers only the part of controlling the load, frequency and the turbine exhausted temperature. The frequency of the generator is presented to the turbine controller through three channels; the average value of these three is selected via 1-out-of-3 logic and considered to be the actual frequency for frequency or the speed for the controller. The load set point is amendable within certain limits by the operation and monitoring (OM) system for the purposes of coordinating unit load. By the OM system, the mode of control can be selected whether power is to be controlled in load operation by speed controller or in load operation by load controller. This regulates the gas given to the turbine for the required production of power. Natural gas consumption is measured by flow-meters installed upstream of the terminal point of supply. The NG premix control valve is positioned by the valve lift controller of the natural gas premix.

The valve lift is read directly into the gas turbine controller. The pilot gas valve position is changed by the valve lift controller of the pilot gas. Both valves have electro-hydraulic actuators which are operated via two hardware outputs to the two coils of the electro-hydraulic actuators. Undesirable compressor operation is prevented via the compressor pressure ratio limit controller (also known as π controller). The function of the cool air limit controller is to rule out mode of operations, which leads to inadequate flow of cooling air to the turbine blades. The system exhausted temperature is being controlled by the IGVs by varying the air mass flow into the combustion chamber. Exhausted temperature is measured immediately downstream of the gas turbine via 24 triple-element thermocouples (MBA26CT101A/B/C to MBA26CT124A/B/C) placed around the surroundings of the exhaust diffuser. All B and C signals from the 24 triple-element thermocouples are used to calculate the mean turbine outlet temperature. These IGVs signal is influenced, in such a way, by two signals: one from the exhausted temperature control and the other from the compressor pressure ratio limit controller (Daewoo E&C, Siemens 2009 Approval). The portion of interest of the automation unit is described and the next section presents the proposed upgrade of the control system for the purpose performance enhancement.

### Generalized predictive controller (GPC) design and implementation

Model predictive control is a well recognized control system technology for controlling power plants and many industrial processes (Bittani and Poncia 2003; Mohamed et al. 2012, Oluwande 2001; Badgwell and Qina 2003). Although there are many other modern control techniques in the previously published literature (Lee and Ramirez 2001), state space formulation of multivariable model based predictive control has been selected for this specific application for so many reasons. First of all, the practical constraints of the control signals and the output signals of the model can be easily considered in the computation algorithm of the controller. In addition, the influences of noises that satisfy the nature of power plant can be included in the control system responsibility. Finally, the world leading electric utilities use this technology in power plant control. The use of MPC has been justified. In addition, the simplicity of using linear MPC with considerations of noises and disturbances is valued over complexity of using nonlinear model predictive control based on deterministic nonlinear model. This is because of the higher computation demands of nonlinear MPC which has lead to its rare industrial applications in comparison to linear state space MPC (Mohamed et al. 2012). A model based predictive control is developed with provisions of unmeasured disturbances and measurement noises to be used for compensation around the investigated operating conditions. Here, the linear time invariant model developed by subspace method in the second section has been used inside the model prediction algorithm. However, many models are developed beforehand and tested by comparison with each other for the one which gives the most feasible controller performance. Thereafter, the model has been augmented as follows:

19 $$x(k + 1) = Ax(k) + B_{u} u(k) + B_{v} v(k) + B_{w} w(k)$$

20 $$\begin{aligned} y(k) & = y(k) + z(k) \\ & = Cx(k) + D_{u} u(k) + D_{v} v(k) + D_{w} w(k) \\ \end{aligned}$$

where *v* is the measured disturbance and *w* is the unmeasured disturbance vector, *z* is the measurement noise. The adopted predictive control algorithm is quite analogous to Linear Quadratic Gaussian procedure (LQG), but with implication of the operational constraints. The prediction is made over a specific prediction horizon. Then, the optimization program is executed on-line to calculate the optimal values of the manipulated variables to minimize the objective function below:

21 $$\xi (k) = \sum\limits_{{i = H_{w} }}^{{H_{p} }} {\left\| {y(k + \left. i \right|k) - r(k + \left. i \right|k)} \right\|} Q + \sum\limits_{i = 0}^{{H_{c} - 1}} {\left\| {\Delta u(k + \left. i \right|k)} \right\|}^{2} R$$

The weighting coefficients (Q and R), control interval (*Hw*), prediction horizon (*Hp*) and control horizon (*H*_*C*_) of the performance objective function will affect the performance of the controller and computation time demands. The terms *r* represents the demand outputs used as a reference for MPC model and Δ*u* is the change in control values for *HC* number of steps. Zero-order hold method is then used to convert the control signal from discrete to continuous fed to the plant.

The constraints of inputs are expressed as minimum and maximum permissible inputs,

22 $$u_{\rm min } \le u \le u_{\rm max }$$

23 $$\Delta u_{\rm min } \le \Delta u \le \Delta u_{\rm max }$$

The control system optimized signal is generated by the control law,

$$\mathop {\hbox{min} \xi (k)}\limits_{{\Delta u, \ldots \Delta u(k + 1 + H_{c} )}} \,{\text{Subject to }}\left( { 22} \right){\text{ and }}\left( { 23} \right)$$

**Fig. 9:**
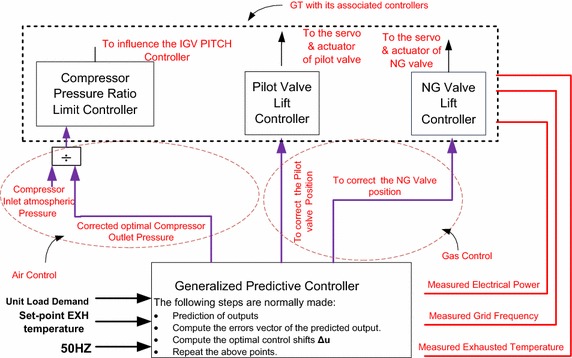
The integration of model predictive control into the associated control of the GT

Traditionally, quadratic programming (QP) solver is used, with interior point method or active set method, to solve the optimization problem of the MPC. The package of the proposed system is shown in Fig. 9. A quantified description of the upgraded strategy of control should be given in words. In the proposed strategy, one important signal is the NG valve position reference necessary to supply the fuel energy to the combustion chamber and satisfy the concept of energy balance in plant thermodynamics. The second signal is the pilot gas valve position reference which is very important to stabilize the premix flames. The third is the best compressor pressure ratio that is corrected by the MPC and fed into the compressor pressure ratio limit controller and eventually will have a positive impact on the IGV pitch controller, compressor actual outlet pressure, and the necessary air flow. Thereby, it is supplying higher amount of air flow to the combustion chamber and reduces the fuel consumption and finally improves the efficiency. Great pressure ratios may cause compressor surging; however, there will not be any such problems because the practical safe limits or constraints of the pressure ratio are naturally included in the MPC optimization algorithm and can be limited by the pressure ratio limit controller. The integrated system is tested in the next section by simulations on a personal computer environment.

## Simulation results

**Fig. 10:**
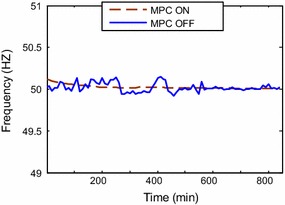
Frequency response

**Fig. 11:**
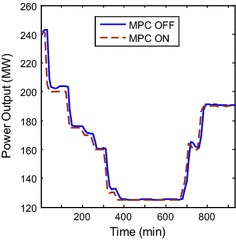
Power response

**Fig. 12:**
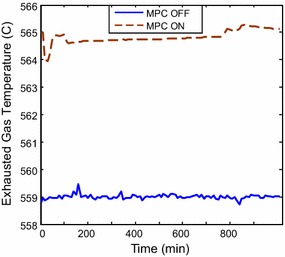
Exhausted temperature responses

**Fig. 13:**
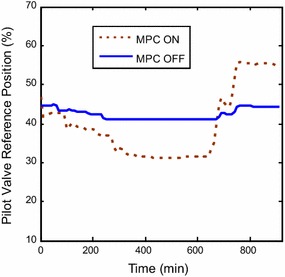
Pilot valve position response

**Fig. 14:**
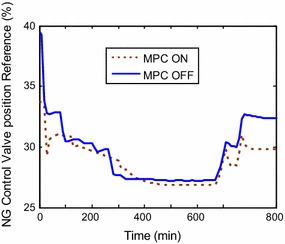
NG valve response

**Fig. 15:**
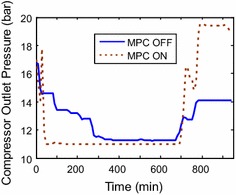
Compressor outlet pressure

**Fig. 16:**
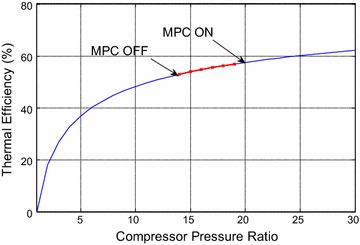
Thermal efficiency for the GT cycle with including the efficiency for period 1

**Fig. 17:**
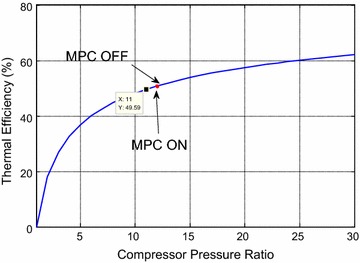
Thermal efficiency for the GT cycle with including the efficiency for period 2

**Fig. 18:**
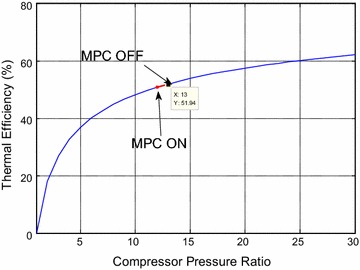
Thermal efficiency for the GT cycle with including the efficiency for period 3

MPC tuning is finalized by selecting appropriate values for the prediction horizon *Hp*, control horizon *HC*, and weighting matrices **Q** and **R.** The control interval, prediction, and control horizons are found to be 1, 40, 5 s respectively. **Q** = [1 1 1] and **R** = [0.2 0.2 0.2]. Simulating different scenarios have caused this selection. In this scenario, a load demand signal extracted from the data during classical closed loop control is used as one of the set-point signals injected to the MPC, with higher exhausted temperature set-point of 565C^Ɵ^ and the frequency should be maintained at 50 Hz. From simulations, it can be readily seen that when the plant control strategy is integrated with model predictive control (MPC ON state); the frequency response is smoother than that case of MPC OFF (Fig. 10). Less frequency variations are found in case of MPC, however, that should be also expected from the power response as faster load following means less excursions (Fig. 11). Since gas turbines in general are very sensitive to frequency deviations and cascaded trip may occur in case of large frequency disturbances, it is now assured that the possibility of relay malfunctioning for trip singling is reduced for the gas turbine in case of MPC ON state. The plant has faster load following capability with less settling time as shown in Fig. 11. It is believed that that has been as a result from predicting the control signals of the NG valve, the pressure ratio, and pilot gas valve in advance, which help in achieving stable and rapid combustion. The response of the temperature is depicted in Fig. 12; higher temperature is maintained during system operation, which means higher thermal energy is supplied to the HRSG. However, this set point can also be amended by the operator in case of changes of ambient conditions to set the suitable reference temperature for the exhausted gas. The proposed signals of manipulated variables have relatively different trends in comparison with classical control system. These corrected signals are amended by the optimization routine in the MPC and can be seen that they are feasible and within the operating restrictions reported by the plant manufacturer. Figures 13 and 14 show the fuel preparation signals for the demanded dynamic set points, it is seen that the MPC has given constrained signals within shorter time of periods. From the signal of the outlet compressor pressure in Fig. 15, the pressure ratio is high in case of high load demands with (MPC ON) state; Therefore, higher GT efficiency during large load operating regions which the plant is very likely to operate. As an estimate of how much improvement has been achieved, the reader is recommended to inspect the thermodynamic curves of pressure ratio depicted in Simões-Moreira (2012) and Ibrahim and Rahman (2012). It can be deduced that upgrading control system with the MPC has promising performance with regard to better system operation and improved responses. This can be accurately expressed in the following points: firstly, the general curve relating the pressure ratio and overall thermal efficiency is redrawn using MATLAB. Second, the pressure ratio is found from the signals arrays of compressor pressure outlet to the atmospheric pressure while the total efficiency (the ratio of the total work done by the CCGT unit to the heat input) is given by thermodynamic relations with the pressure ratio, the equation is used to plot the curve of thermal efficiency for three distinct periods in which the efficiency including MPC may be higher or lower than that without the MPC. The approach used to calculate the average efficiency for the working hours is very simple. The compressor outlet pressure response is used to find the pressure ratio which is used to plot the efficiency. Then, the average efficiency is found for three different periods of operating the plant. Curve 1 is shown in Fig. 16. It can be readily seen that the red part of the curve represents the deviation in efficiency in the from minute 700 to minute 950 with around (250 working minutes), the compressor outlet pressure (and hence the pressure ratio for 1 bar atmospheric pressure) raised from 14 to 19.3 with (MPC ON) state in comparison with the normal strategy (MPC OFF) with corresponding efficiencies of (52.9–56.88 %), respectively (or 3.98 % increase in efficiency). Another investigated period of operation is shown in (Fig. 17). There is a small decrease in the compressor outlet from 11.7 to 11.5 that corresponds to efficiencies of (49.7–50 % = −0.3 %). Similarly, in Fig. 18, there is a decline in the compressor outlet pressure from 13 to 11.5 bars from the existing to the new strategy; However, this will result in decreasing the efficiency from 51.8 to 49.9 % (i.e. 49.9–51 = −1.1 %). The average thermal efficiency for three equal intervals of operation (η_av_ = (3.98 – 0.3 – 1.1)/3 = 1.1 %). However, this argument can also be supported from time based simulations. NG control valve position is also an obvious indicator for reduced fuel consuming (Fig. 14). The plant situation with integration of MPC is more improved than existing control strategy without MPC and more suitable for satisfying the grid obligations.

## Conclusions

A feasible application of model predictive control into GT air and its associated controls is newly proposed. The suggested configuration acts as corrector for the key controllers and their actuators that affect the system efficiency through the compression ratio, power dynamic response contribution to the grid, and heat sent to the HRSG. Simulation studies have shown encouraging results that stimulates further research and practical implementation. As a future recommendation, it is suggested that the attention is turned to the HRSG for further enhancement to the system performance. Also, it is intended to handle some practical issues that are not undertaken by the model and its associated control system. These are the two points mainly to extend this research:

1. The composition of natural gas produced in Libyan varies from time to another and here are some examples supplied by our industrial partners in Sirte Oil Company for Production and Manufacturing of Oil & Gas, Technical Dept/Process Engineering & Labs Division. The compositions are in two different dates in different years. On 16/02/2011: Methane (81.47 %), ethane (11.15 %), propane (2.7 %), iso-Butane (0.5 %), n-Butane (0.61 %), iso-Pentane (0.19 %), n-Pentane (0.13 %), nitrogen (0.52 %), Carbon Dioxide (2.7 %), Hexane (0.03 %).
  On 19/01/2012: Methane (82.98 %), ethane (6.82 %), propane (1.97 %), iso-Butane (0.38 %), n-Butane (0.48 %), iso-Pentane (0.24 %), n-Pentane (0.16 %), nitrogen (0.41 %), Carbon Dioxide (6.5 %), Hexane (0.06 %). These variations affect fuel calorific value and consequently the efficiency of the plant. However, including all factors that affect the thermal efficiency and/or dynamic responses is quite complex and difficult to achieve in the present industry.
2. The MPC performance is not very ambitious in some small intervals like that mentioned in the previous section, although these small intervals are not likely to remain and the average overall efficiency over 800 min is higher. These limitations in the controller performance can be handled by adapting the control parameters and/or using nonlinear model predictive control. The value of using nonlinear model predictive control is that it handles the uncertainty associated with the nonlinearity of the plant. Thereby, improved control performance but increasing the computation burdens on the centralized computer used for control. This practical issue along with the 1st one are the authors’ interest for the long-term research work.

## Appendix

$$A = \left[ {\begin{array}{*{20}l} { 0. 6 6 2} \hfill & { - 0. 6 2 2} \hfill & {{ - 0} . 0 5 4} \hfill & { 0. 1 0 4} \hfill & { 0. 0 0 4} \hfill & { 0. 1 3 7} \hfill & {{ - 0} . 0 3 7} \hfill & { 0. 0 8 2} \hfill & { 0. 0 2 8} \hfill & { 0. 0 2 6} \hfill \\ { 0. 5 1 8} \hfill & { 0. 0 3 8} \hfill & { 0. 1 1 6} \hfill & { 0. 0 4 6} \hfill & {{ - 0} . 1 6 2} \hfill & { 0. 3 1 3} \hfill & {{ - 0} . 2 0 4} \hfill & { 0. 1 1 3} \hfill & {{ - 0} . 0 4 9} \hfill & { 0. 0 9 2} \hfill \\ {{ - 0} . 3 0 9} \hfill & { - 0.523} \hfill & { - 0.411} \hfill & { - 0.18} \hfill & { - 0.327} \hfill & {0.422} \hfill & {0.413} \hfill & {0.293} \hfill & { - 0.25} \hfill & {0.136} \hfill \\ {0.046} \hfill & {0.117} \hfill & { - 0.682} \hfill & {0.447} \hfill & { - 0.09} \hfill & {0.425} \hfill & { - 0.137} \hfill & {0.438} \hfill & {0.337} \hfill & {0.324} \hfill \\ { - 0.001} \hfill & { - 0.169} \hfill & {0.157} \hfill & { - 0.42} \hfill & {0.506} \hfill & { - 0.262} \hfill & { - 0.117} \hfill & {0.355} \hfill & {0.162} \hfill & {0.551} \hfill \\ { - 0.191} \hfill & { - 0.07} \hfill & {0.15} \hfill & {0.143} \hfill & {0.315} \hfill & {0.989} \hfill & { - 0.284} \hfill & { - 0.298} \hfill & {0.072} \hfill & { - 0.159} \hfill \\ { - 0.007} \hfill & {0.077} \hfill & {0.208} \hfill & { - 0.12} \hfill & {0.029} \hfill & { - 0.274} \hfill & {0.133} \hfill & { - 0.249} \hfill & {0.813} \hfill & { - 0.029} \hfill \\ {0.289} \hfill & {0.347} \hfill & { - 0.385} \hfill & { - 0.614} \hfill & { - 0.153} \hfill & {0.184} \hfill & {0.225} \hfill & {0.039} \hfill & {0.053} \hfill & {0.046} \hfill \\ {0.279} \hfill & {0.218} \hfill & { - 0.46} \hfill & { - 0.039} \hfill & {0.022} \hfill & { - 0.524} \hfill & { - 0.894} \hfill & {0.036} \hfill & { - 0.299} \hfill & { - 0.151} \hfill \\ {0.058} \hfill & {0.06} \hfill & { - 0.008} \hfill & {0.084} \hfill & {0.275} \hfill & {0.505} \hfill & {0.239} \hfill & {0.566} \hfill & { - 0.358} \hfill & {0.367} \hfill \\ \end{array} } \right]$$

$$B = \left[ {\begin{array}{*{20}l} { - 0.378} \hfill & { - 0.167} \hfill & {0.485} \hfill \\ { - 0.367} \hfill & { - 0.242} \hfill & {0.305} \hfill \\ { - 1.053} \hfill & { - 0.463} \hfill & {2.611} \hfill \\ { - 0.784} \hfill & { - 0.343} \hfill & {1.675} \hfill \\ { - 0.051} \hfill & { - 0.219} \hfill & { - 1.084} \hfill \\ {1.055} \hfill & {0.623} \hfill & { - 2.236} \hfill \\ {1.907} \hfill & {1.074} \hfill & { - 4.922} \hfill \\ {3.21} \hfill & {1.796} \hfill & { - 6.506} \hfill \\ {1.435} \hfill & {0.401} \hfill & { - 2.751} \hfill \\ { - 2.971} \hfill & { - 1.53} \hfill & {7.583} \hfill \\ \end{array} } \right]$$

$$D = \left[ {\begin{array}{*{20}l} 0 \hfill & 0 \hfill & 0 \hfill \\ 0 \hfill & 0 \hfill & 0 \hfill \\ 0 \hfill & 0 \hfill & 0 \hfill \\ \end{array} } \right]$$

$$C = \left[ {\begin{array}{*{20}l} { - 32.31} \hfill & {15.18} \hfill & {1.541} \hfill & {2.44} \hfill & { - 14.68} \hfill & { - 21.06} \hfill & {3.322} \hfill & {2.06} \hfill & { - 0.722} \hfill & { - 0.926} \hfill \\ {14.42} \hfill & { - 2.019} \hfill & { - 5.686} \hfill & {3.799} \hfill & {7.479} \hfill & { - 15.42} \hfill & {1.735} \hfill & {1.48} \hfill & {2.418} \hfill & { - 2.35} \hfill \\ {1.33} \hfill & { - 0.2145} \hfill & { - 0.603} \hfill & {0.508} \hfill & { - 0.709} \hfill & { - 1.336} \hfill & {0.186} \hfill & {0.148} \hfill & {0.126} \hfill & { - 0.210} \hfill \\ \end{array} } \right]$$

## List of symbols

*A*: system matrix $$\in R^{n \times n}$$
*B*: system matrix $$\in R^{n \times m}$$
*C*: system matrix $$\in R^{l \times n}$$
*D*: system matrix $$\in R^{l \times m}$$
*E*_*f*_: future noise
*H*_*i*_^*d*^: deterministic Toeplitz matrix
*H*_*i*_^*s*^: stochastic Toeplitz matrix
*H*_*P*_: prediction horizon
*H*_*C*_: control horizon
*Hw*: control interval
*N*_*f*_: future noise
*n*: system order
*O*_*i*_: extended observability matrix
*Q*: system matrix $\in R^{n \times n}$ or weighting coefficient in MPC
*R*: system matrix $\in R^{n \times l}$ or weighting coefficient in MPC
*S*: system matrix $$\in R^{l \times l}$$
*u*_*k*_: input at instant k
**U**: orthogonal matrix
*U*_*f*_: future input
*U*_*p*_: past input
*v*_*k*_: zero mean white noise innovations at instant k
**V**: orthogonal matrix
$$\mathcal{V}$$: subspace
*W*_1_: weighting matrix
*W*_2_: weighting matrix
$$\mathcal{W}$$: subspace
*x*_*k*+1_: system state at instant k + 1
*x*_*k*_: system state at instant k
*X*_*i*_: sequence of states vector
*X*_*f*_: future states
*y*_*k*+1_: system output at instant k + 1
*y*_*k*_: system state at instant k
*Y*_*f*_: future output
*Y*_*p*_: past output
*ξ*(*k*): objective function to be minimized by the MPC
